# Do Health Information Sources Influence Health Literacy among Older Adults: A Cross-Sectional Study in the Urban Areas of Western China

**DOI:** 10.3390/ijerph192013106

**Published:** 2022-10-12

**Authors:** Chengbo Li, Mengyao Liu, Jin Zhou, Mei Zhang, Huanchang Liu, Yuting Wu, Hui Li, George W. Leeson, Tingting Deng

**Affiliations:** 1 School of Journalism and Communication, Chongqing University, Chongqing 400044, China; 2 Oxford Institute of Population Ageing, University of Oxford, Oxford OX2 6PR, UK

**Keywords:** health literacy, sources of health information, interpersonal communication, mass communication, older Chinese adults

## Abstract

Background: Previous studies have found that the dissemination pattern and delivery mechanism of information can provide crucial resources and empowerment for individuals to the promotion of health literacy. The present study investigates how health information sources are associated with health literacy among older adults in west China, and tries to explain the mechanisms underlying the link between health information sources and health literacy in the Chinese context. Methods: The cross-sectional study employed a representative sample of 812 urban citizens aged 60 and older in 2017 in Western China. Results: We found that health information sources including healthcare practitioners (B = 4.577, *p* < 0.001), neighbors (B = 2.545, *p* < 0.05), newspapers (B = 4.280, *p* < 0.001), and television (B = 4.638, *p* < 0.001) were positively associated with health literacy. Additionally, age (B = −1.781, *p* < 0.001) was negatively associated with health literacy, and the socio-economic status factors including minority (B = −10.005, *p* < 0.001), financial strain status of perceived very difficult (B = −10.537, *p* < 0.001), primary school (B = 11.461, *p* < 0.001), junior high school (B = 18.016, *p* < 0.001), polytechnic school or senior high school (B = 21.905, *p* < 0.001), college and above (B = 23.433, *p* < 0.001) were significantly linked to health literacy, and suffering from chronic diseases (B = 3.430, *p* < 0.01) was also positively related to health literacy. Conclusions: Health information sources including healthcare practitioners, neighbors, newspapers, and television have a strong influence on health literacy, which implies that the four main types of sources are the important patterns of health information dissemination in the reinforcement of health literacy. In addition, the present findings also indicate age, minority and disease differences in health literacy and confirm the influence of enabling factors including educational attainment and financial strain on health literacy. Based on these findings and their implications, specific evidence is presented for the reinforcement of health literacy in interpersonal and mass communication, and in the educational and financial settings in the Chinese context. The present results also suggest that the age-specific, minority-specific and disease-specific measures should be taken to promote health literacy among older adults.

## 1. Introduction

Health literacy means that the ability to obtain, understand and use health-related information and services in ways that lead to reasonable health decisions [1,2]. Health literacy represents and implies a fundamental knowledge, skill and confidence in enhancing health for the individual and community. Thus, health literacy is critical to the participation and empowerment of people and community [1]. Moreover, health literacy is an important health priority [3] and one pillar of health promotion [4,5] at local, national and global level.

Health literacy is an increasing global concern [6,7]. First of all, the initiatives and actions of health literacy reinforcement can contribute to a health literate organization and society, and ultimately help people, in particular older adults, maintain health literacy and lead longer, healthier lives [8,9]. In China, the reinforcement of health literacy is a crucial precondition for national health promotion [10]. The goals in healthy China strategies [11], healthy China action (2019–2030) [12] and China’s scientific literacy action (2021–2035) [13] highlight the importance of promoting health literacy for all people, especially for older individuals. What’s more, adequate health literacy is important for understanding health-related information and risks [14], so that healthy behaviors and lifestyles can be engaged [15]. Furthermore, it is vital to have sufficient health literacy to improve self-efficacy [16], reduce the hospitalization rates [17], and for the elderly to make good health decisions [18].

Most of the elderly have one or more chronic diseases, and they often have difficulty in dealing with their healthcare issues [19]. Lack of health literacy and its negative effect would exacerbate such difficulties and result in less self-management, various of health risk behaviors and adverse health outcomes [20,21]. Moreover, China has one of the largest and fastest growing older population across the world, and the results from census 2020 have shown that the size of the usual resident population aged 60 and older was 264.02 million in China [22]. In the context of China’s population aging, the elderly’s poor health literacy could challenge the promotion of health literacy for all people, and an increasing of financial burden and disease burden in the future [23].

Most of the elderly are a particularly disadvantaged population with inadequate health literacy [3,24,25,26] in a many countries, such as the U.S. [27], Germany [28], and other certain European countries including Austria, Bulgaria, Greece, and Spain [29], and in Ontario, Canada [30]. In China, inadequate health literacy was also identified, the data indicated that the prevalence of adequate health literacy for the elderly and urban elderly aged 65~69 was 8.49% in 2020 [31] and 8.55% in 2019 [32] at national level, respectively. In addition, one study with meta-analysis also found that the prevalence of adequate health literacy was 12.28% for the elderly in China in 2021 [33], while 23.15% of the national residents aged 15–69 had adequate health literacy in 2020 [31].

Based on the investigation in 2017 from China’s health education center, only 6.42% of the older people aged 60–69 had adequate health literacy in Western China, while the adults aged 15–69 were 10.96% in China [34]. There was a significant disparity between Western China and China. Hence, it is essential and urgent to take action to improve health literacy for older population in Western China [35]. Moreover, some studies have demonstrated that the dissemination pattern and delivery mechanism of health information can provide crucial resources and empowerment for individuals to the promotion of health literacy [18,36,37,38]. Most of the people could benefit from the number of information sources used [39,40,41] when finding health information and making health decisions. Moreover, access to information from interpersonal communication [42] and mass communication [43,44] can contribute to improving health literacy. Our current study tries to confirm this view, and determine whether health information sources could influence health literacy among older adults in the urban areas of Western China.

Furthermore, there was a socially contextualized consideration of health literacy [45,46,47]. Health literacy was not considered an individual trait [45], and it referred to using individual’s social resources to achieve health goals in the World Health Organization’s definition [48] and many international settings [49]. In addition, previous studies have consistently indicated the role of social context in health literacy [50,51,52,53]. Therefore, from the social context perspective, our current study also tries to examine whether health literacy is sensitive to the social context among older Chinese adults.

## 2. Literature Review

### 2.1. The Association between Health Information Sources and Health Literacy

Studies have demonstrated the strong association between health information sources and health literacy. The literature has stated that participants those who have more sources of health information [18,38] and better access to health information [37] have a greater likelihood of being health literate. In addition, research has found that obtaining health information from health professionals or the internet contributes to health literacy in the U.S. [36].

The mass media play a constructive role in the reinforcement of health literacy. Mass communication can support health literacy in many ways, and most Europeans seek out supplementary information by mass media [4]. Research has shown that receiving health information from books or magazines is positively associated with health literacy [18]. Research indicates that the mean health literacy score increases as the number of books and newspapers read in a year increases in Edirne, Turkey [54]. Moreover, in Vietnam, older individuals with adequate health literacy benefit from obtaining health information through radio, TV and newspapers [55].

The internet is instrumental in facilitating health literacy. Using the internet for health information can contribute to health literacy among older adults in Vietnam and the U.S. [18,36,55]. Research reveals that those who have higher averages in health literacy would be more likely to seek for health information through health professionals and the internet in the U.S. [36] and in Rio Grande-RS, Brazil in 2017 [56]. Older adults and those who have less access to health information through the internet are less likely to be health literate [57]. One study discovered that using the internet for health information is effective in increasing the health literacy level among the elderly in the U.S. [58].

The majority of participants when confronted with a serious health issue would turn to friends and family for health information or support [59,60]. In the Netherlands, family members often share health information, and provide informational support, which could contribute to the elderly’s health knowledge and literacy [61].

Healthcare practitioners could help older adults maintain adequate health literacy skills. Healthcare professionals are considered as the primary and most trusted information source of health information among older adults in Europe [4], the U.S. [60,62], and Switzerland [63]. Previous study findings indicated that health literacy tightly linked to obtaining information from healthcare providers in the U.S. [36] and Thailand [37].

Neighbors as interpersonal sources of information can contribute to the elderly’s health literacy. Neighbors can serve as role models of behavior change and contribute to better health outcomes, as Pechrapa et al. said “When a neighbor has good health literacy, the older adults will also have good health literacy [37]”.

### 2.2. Other Factors Related to Health Literacy

Past studies have examined other predictors of the elderly’s health literacy. The other factors include demographic characteristics, socio-economic status, and health status.

Empirical studies have investigated the demographic predictors of health literacy. Health literacy declines with age among older individuals [55,64,65,66]. For instance, a study has revealed that sufficient health literacy is prevalent among young people, while insufficient health literacy is predominant among older people in Ghana and the U.S. [67,68]. Moreover, some research has not supported the association between age and health literacy in Canada [30] and Finland [69]. Research has indicated a higher health literacy for older men in Iran [70] and the U.S. [71], however, some research has also indicated a lower health literacy for older men in the UK [72].

Studies have discovered that poor health literacy is prevalent among the elderly with lower socio-economic status. For instance, a minority group is far less likely to be health literate among the elderly, and black participants or ethnic minorities typically have poorer health literacy compared to the white population of older adults in the U.S. [68,73]. Educational attainment positively affects health literacy among older adults [15,18,30,56,68,71] and is particularly linked to the elderly’s health literacy in China [33] and Turkey [74]. Additionally, occupation is shown to be linked to health literacy, so that older adults of a low occupational class are vulnerable population with limited health literacy in England [72], Vietnam [55] and South Korea [75]. Older participants who are famers show a faster decline in health literacy than those who are non-farmers in China [33]. Financial strain is a strong predictor of health literacy [15,18,69,76], specifically, older adults with lower income show a faster decline in health literacy in the U.S. [71,77] and Brazil [56].

Having better physical health is an important determinant of greater health literacy, as is self-rated health in China [33], Ghana [67] and Finland [69]. Moreover, many studies have indicated respondents with multiple chronic conditions are more likely to be health illiterate [2,69,78,79], while other research has found that suffering from chronic diseases is related to sufficient health literacy and this relationship is likely to strengthen as the number of chronic conditions increased [80].

The literature reviews above indicate that few studies have focused on investigating the link between health information sources and health literacy, and the in-depth explorations of the association are lacking. These disparities highlight the importance of in-depth exploration of the association in China. Therefore, the present cross-sectional study aims to focus on observing the influence of health information sources on the elderly’s health literacy in the urban areas of Western China.

## 3. Research Method

### 3.1. Data

The present study and the work of Li et al. [81,82] used the same data. More details regarding the sampling design, process of survey sampling and data collection were stated below.

In 2017, we implemented a cross-sectional survey in urban areas of Western China, and the sampled regions included Yinchuan of Ningxia, Wenshan of Yunnan, and Yongchuan of Chongqing. From July to September, 812 urban older adults aged 60 and above were selected by using a stratified multistage and cluster random sampling design.

The process of survey sampling was divided into five stages. First, the three provinces or municipalities including Ningxia, Yunnan and Chongqing, were selected in Western China. Second, the three cities including Yinchuan, Wenshan, and Yongchuan were selected from Ningxia, Yunnan, and Chongqing, respectively. Third, one urban block in Yinchuan, Wenshan, and Yongchuan was randomly selected, respectively. Fourth, in total, 33 urban residential communities were randomly selected, 5 from Yinchuan, 16 from Wenshan, and 12 from Yongchuan. Fifth, based on the selected urban residential communities, 812 households were randomly selected, 203 from Ningxia, 278 from Yunnan, and 331 from Chongqing. If there was more than one person aged 60 or older in one household, we would use the Kish table to randomly select one individual.

The data collection had several specific steps. First, the written consent regarding the purposes and objectives of this study were provided before the implementation. Second, using paper-assisted and face-to-face personal interviews, we issued and implemented health literacy survey questionnaires when the interviewees agreed to participate. Finally, a total of 812 questionnaires were collected and the response rate was 100% (812 of 812).

### 3.2. Measurement Instruments

The literature reviews of our present study have provided sufficient references to support the measurement of the possible factors. The possible independent variables consisted of health information sources (healthcare practitioners, family members, neighbors, community workers, newspapers, magazines and books, television, and internet), the possible controlled variables consisted of three factors: demographic characteristics (gender and age), socio-economic status (ethnicity, educational attainment, occupation and financial strain) and health status (self-rated health and suffering from chronic diseases).

#### 3.2.1. Health Literacy

More details about the measurement of health literacy were mentioned in Li et al. [82]. Health literacy was assessed using the Chinese version of health literacy questionnaire, which has been widely applied and confirmed in the past studies [23,83,84,85,86,87] and could ensure good reliability and validity. Our current study adopted this version, which was developed by China Health Educational Center. In total, there were 80 items covering three aspects in the health literacy questionnaire. The three aspects of health knowledge, behaviors and skills consisted of 38 items, 22 items and 20 items, respectively. In our sample, Cronbach’s alpha for health literacy was 0.937. More details about the 80 items of health literacy were stated in Nie et al. [87] and Li. [86].

The format of all of the test items was in the form of four types of questions: true-or-false questions, single-answer questions (a score of one for a correct answer and a score of zero for an incorrect answer), multiple-answer questions (a score of two for all correct answers and a score of zero for all incorrect answers) and situation questions in the form of reading comprehension questions regarding common information, instructions and knowledge related to medicine and health in everyday life, which included single-answer questions and multiple-answer questions (with the same scoring criteria as the single or multiple-answer questions described above). In each item, an answer of “I don’t know” was given a score of zero. The scores from the 80 items with equal weighting were summed up to create an overall score [87,88], and the range of scores for health literacy was 1–94 in the current study. 

#### 3.2.2. Health Information Sources

Health information sources were measured in the form of multiple-answer questions. Health information sources were classified into eight main items: healthcare practitioners, family members, neighbors, community workers, newspapers, magazines and books, television and internet. Participants were asked whether they had used one or more sources for getting health information. Respondents were asked to rate each item on a two-point scale (0 = no, 1 = yes).

#### 3.2.3. Other Variables

The other variables included three factors: demographic characteristics, socio-economic status, and health status.

Demographic information mainly included gender (0 = male, 1 = female) and age (1 = 60–64, 2 = 65–69, 3 = 70–74, 4 = 75–79, and 5 = 80+).

Socio-economic information mainly consisted of ethnicity (1 = Han, 2 = minority), educational attainment (1 = illiterate, 2 = primary school, 3 = junior high school, 4 = polytechnic school or senior high school, 5 = college and above), occupation (1 = ordinary staff, 2 = professional, 3 = manager, 4 = service industry employee, 5 = production staff, 6 = other), and financial strain, which was measured by a question: “How do you assess and report your finan-cial status now?” Each response was rated on a 5-point Likert scale (1 = more than enough, 2 = good enough, 3 = approximately enough, 4 = somewhat difficult, 5 = very difficult).

The two variables self-rated health and suffering from chronic diseases were regarded as the measurement of health status. Self-rated health was measured by a single item: “How do you assess and report your health situation now?” Each response was rated on a 3-point Likert scale (1 = bad, 2 = fair, 3 = good). Suffering from chronic diseases was assessed by a question: “Do you suffer from any chronic disease?” Each response was rated dichotomously (0 = no, 1 = yes).

### 3.3. Data Analysis

By applying IBM SPSS 22.0 software (SPSS Inc., Chicago, IL, USA), we used descriptive statistics and a multiple linear regression model to observe the influences of the selected factors on health literacy. Moreover, we set this sample’s statistical significance at 0.05. First, descriptive statistics were applied to calculate the frequencies (percentages), means (standard deviations) and *F*/*t*-test results for all variables. A *F*/*t*-test was used to compare the means of health literacy by different characteristics. Second, a multiple linear regression model was used in the present sample, and the regression non-standardized coefficients (B), standardized coefficient(β), confidence intervals (95% CI) and *p*-values were reported.

Based on the reviewed literature and research method above, the two equations for the multiple linear regression are:

Model I: *y_i_* = *β*_0_ + *β_i_ X_i_* + *ε_i_* (*i* = 1–8)

Model II: *y_j_* = *γ*_0_ + *β_j_ X_j_* + *ε_j_* (*j* = 1–16)

*y_i_* meant that the dependent variable health literacy in Model I.

*y_j_* meant that the dependent variable health literacy in Model II.

*X_i_* meant that the 8 independent variables consisted of healthcare practitioners, family members, neighbors, community workers, newspapers, magazines and books, television, and internet.

*X_j_* meant that the 16 variables, including the 8 independent variables (healthcare practitioners, family members, neighbors, community workers, newspapers, magazines and books, television, and internet) and the 8 controlled variables (gender, age, ethnicity, educational attainment, occupation, financial strain, self-rated health and suffering from chronic diseases).

*β*_0_ referred to *y_i_* intercept (constant term) in Model I, *γ*_0_ referred to *y_j_*-intercept (constant term) in Model II.

*ε_i_* meant that the error term (also known as the residuals) in Model I.

*ε_j_* meant that the error term (also known as the residuals) in Model II.

## 4. Results

### 4.1. Characteristics of the Sample

We could find from Table 1 that the average score for health literacy was 56.32 (SD: 18.44). Additionally, the percentages of receiving health information were healthcare practitioners (86.3%), television (55.9%), family members (54.7%), neighbors (27.0%) and newspapers (19.0%), respectively.

### 4.2. Multiple Linear Regression Model

Before applying the regression models, multicollinearity was checked among all independent variables. The results showed that all of the tolerance values of the independent variables were greater than the common threshold of 0.1, indicating that multicollinearity was at an acceptable level. Moreover, all of the VIF in the table were less than 5. Therefore, there was no multicollinearity issue in the regression.

A multiple linear regression model I—with all eight health information source indicators introduced as independent variables—explained 25.9% of the variance in health literacy (Table 2). We found that healthcare practitioners (B = 6.180, *p* < 0.001), family members (B = 3.395, *p* < 0.01), and neighbors (B = 4.564, *p* < 0.001), newspapers (B = 10.577, *p* < 0.001), television (B = 9.510, *p* < 0.001), internet (B = 9.287, *p* < 0.001) were significantly and positively associated with health literacy. Specifically, the average health literacy for participants who obtained health information through healthcare practitioners, family members, neighbors, newspapers, television and the internet were 6.180, 3.395, 4.564, 10.577, 9.510 and 9.287 higher than for their counterparts, respectively.

Moreover, by applying a new multiple linear regression model and controlling other factors, such as demographic attributes, socio-economic status and health status, we could conduct a better examination of the influence of the eight health information source indicators on health literacy.

As shown in Table 3, the multiple linear regression model II was adjusted to include other factors to examine whether health information sources were still significantly associated with health literacy. The variables used in the regression analysis explained 57.9% of the variance for participants in terms of health literacy, which indicated strong predictive power.

Among the participants, healthcare practitioners (B = 4.577, *p* < 0.001), neighbors (B = 2.545, *p* < 0.05), newspapers (B = 4.280, *p* < 0.001), television (B = 4.638, *p* < 0.001) were still related to health literacy. In addition, age (B = −1.781, *p* < 0.001), minority (B = −10.005, *p* < 0.001), primary school (B = 11.461, *p* < 0.001), junior high school (B = 18.016, *p* < 0.001), polytechnic school or senior high school (B = 21.905, *p* < 0.001), college and above (B = 23.433, *p* < 0.001), financial strain status of perceived very difficult (B = −10.537, *p* < 0.001), and suffering from chronic diseases (B = 3.430, *p* < 0.01) were also closely associated with health literacy (Table 3). Specifically, the average health literacy for participants who obtained health information through healthcare practitioners, neighbors, newspapers and television was 4.577, 2.545, 4.280 and 4.638 higher than for their counterparts, respectively. A 5-year increase in age resulted in an average 1.781 decrease in health literacy. The average health literacy for minorities were 10.005 lower than for Han Chinese. The average health literacy for participants who had primary education, junior high education, polytechnic or senior high education, college and above was 11.461, 18.016, 21.905 and 23.433 higher than for participants who had illiterate education, respectively. The average health literacy for respondents who reported financial strain status of “perceived very difficult” was 10.537 lower than for respondents who reported financial strain status of “more than enough”. The average health literacy for participants who were suffering from chronic diseases was 3.430 higher than for their counterparts.

## 5. Discussion

This study revealed that health information sources including healthcare practitioners, neighbors, newspapers, and television were strongly linked to health literacy. Consistent with previous study [4], we found that as enabling factors, personal sources of information (healthcare practitioners, neighbors) and media sources of information (television, newspapers) could support and increase health literacy. The reason for the enabling influence is that personal sources and media sources of information can be used as resources for health knowledge and health literacy. When health information from media and interpersonal networks are paid more attention, they can enhance health concepts, lead to an increased health knowledge [89], and provide guidance for people [90,91]. Moreover, the discussion here could provide specific evidence for the reinforcement of health literacy in interpersonal communication and mass communication in the Chinese context.

Consistent with previous study in U.S. [60], we find that healthcare practitioners (86.3 percent) and neighbors (27 percent) are ranked as an extremely important source of health information in our current study, and obtaining health information from healthcare practitioners and neighbors can contribute to enhance health literacy. This finding is consistent with previous study in Canada, which indicated that healthcare practitioners and neighbors as a vital pattern of interpersonal communication or social network communication for learning might develop important health literacy skills [2]. Moreover, previous studies have also identified the association between interpersonal communication and health knowledge acquisition [92,93]. In health communication, interpersonal networks and sources may enable people to gain new information, insights, and perspectives [94,95], and thus more health knowledge [96,97]; in turn, they can encourage people to engage in health behavior changes [89,98,99]. In health promotion, past research demonstrated that interpersonal communication was an effective communication tool [100], and had some advantages [101] and potentials [102] for health promotion.

Specifically, the present study findings showed that healthcare providers had the strongest influence on health literacy. Healthcare providers were the primary and most trusted information source of health information among older adults in Europe [4] and Switzerland [63]. The significant association between healthcare providers and health literacy were confirmed in past research from the U.S. [36], Thailand [37] and Rio Grande-RS, Brazil in 2017 [56]. Moreover, neighbors as a reinforcing factor can enhance health literacy. In Asian countries, neighborhoods support was important to older adults [103,104,105]. Even when most older adults were living with their family, they had free time to spend with their neighbors in the community [37], while health information was often communicated through neighbors, thus, older adults could benefit from neighborhoods support and community resources in enhancing health literacy [4]. What’s more, neighbors and healthcare professionals as the two main types of social support could help older adults to prevent social isolation and moderate stressors on health [106], and then improve the ability to make health decisions in their community context [107,108,109].

Our current study identified that newspapers and television as sources of health information could reinforce health literacy. There were three possible reasons for the association between media sources (newspapers, television) and health literacy. First of all, this might be because newspapers and television were trusted sources of health information in China, and Chinese people were dependent on them for precise information. Meanwhile, newspapers and television as common patterns of mass communication were also important sources of health information; the frequent newspapers and television users were well-informed about health problems [110], and mass media could strengthen the individual’s health knowledge and awareness [111]. Thus, the mass media might have a vital impact on health education, and it has the potential to influence health knowledge for the public. Furthermore, due to the social functions of mass media, it could provide mediated social environments that contain a variety of positive information support and resources, which might assist older adults in assessing and understanding medical conditions [112], and dealing with the healthcare system. Secondly, this association might be because newspapers and television could provide materials and resource to practice reading comprehension related to health issues, and then facilitate and develop health literacy. This finding was consistent with specific evidence from the Europe [4], which revealed that mass communication can support users’ health literacy. Thus, public health and other sectoral authorities and advocates had considered using mass media to strengthen health literacy as strategic approaches, and had taken actions in traditional media settings.

Thirdly, our current findings indicated that television was able to reach the second largest population (55.9 percent). This association also might be because television could cover most of the population [113] and encourage healthy behavior [114,115,116,117]. Data also indicated that China’s television penetration rate had reached 89.17% as early as 2002, higher than the average rate of the middle-income and low-income countries [118]. Moreover, the coverage rate of television had reached 99.07 percent among the population in China in 2017 [119]. In addition, the close link between newspapers and health literacy might be explained by a higher literacy rate in urban areas of China. As the seventh national population census in China in 2020 showed, the literacy rate was 94.81% among urban older adults aged 60 and over [120].

It is worth noting that the link between the internet and health literacy was not identified in the current research. This might be explained by the age-related digital divide. The lack of perceived need [121], interest or motivation [122], knowledge and access [123], and the physical limitation [122] have brought major challenges to use computer [124] and the internet, and then led to diminished use of the internet [125] and digital divide among older adults. Digital divide is a serious issue among older adults in China [126]. Furthermore, older adults are dealing with a digital divide when accessing health information and medical services [127,128]. Most of the older people access information from mass media such as television [129] and family members [41], while fewer elderly Japanese obtain information through the internet compared to the young counterparts [130]. Moreover, existing research shows that older adults have less trust in the internet and information online [131].

Moreover, the current results also indicated the influence of controlling variables on health literacy. The significant controlling variables consisted of age, minority, educational attainment, financial strain status of “perceived very difficult”, and suffering from chronic diseases.

Age was inversely linked to health literacy, consistent with previous studies, people who were older were more likely to experience health literacy decline [72], and be predisposed to low health literacy [132]. This might be because as age increased, cognitive ability declined, leading to decreased ability to learn health knowledge, and to access, understand, appraise, and apply health information [65,133], and then health literacy declined and low health literacy appeared to increase with age.

The influence of the socio-economic factor including ethnicity, educational attainment, and financial strain on health literacy was identified in our current study. This finding indicated the presence of a social gradient in the elderly’s health literacy in Western China. Similar evidence had been found in Europe [29], Australia [134], England [135,136], the U.S. [137] and Switzerland [138].

Ethnic minorities were less likely to be health literate than Han Chinese. This might be because the minority group were particularly vulnerable to literacy decline among the elderly [72]. Similar views were identified in prior research from Tibet [139] and Jilin province [140] of China. Due to socio-economic status challenges, especially the lower educational attainment, ethnic minority elderly were at risk for poor health literacy [141,142].

In the current study, as educational attainment increased, health literacy gradually increased. A higher level of formal education was an important determinant of adequate health literacy in prior studies [25,143,144]. The people who were more educated were more likely to have sufficient health literacy because the status of “more educated” would mean more practices and learning resources to promote health literacy [2]. The present study also confirmed the negative association between the financial strain status of “perceived very difficult” and health literacy. This might be because poor financial status would mean disadvantaged access to financial resources [2], which adversely affect the establishment and the improvement of health literacy [139]. Moreover, individuals’ good educational and financial status are the social context factors that could help to develop health-related knowledge and capability among older adults.

Consistent with a previous study [80], another finding of the present study indicated that those who suffered from chronic disease were more likely to be health literate. First, this might arise because suffering from chronic diseases may act as a need factor to health literacy [2]. Most of the elderly were a vulnerable population with poor physical status [65] and chronic health conditions [145]. Due to ill health and multiple chronic diseases, they would increase the need to seek and understand as much health information as possible about their diseases [146,147,148,149], along with the association between these diseases and their recognition and treatments [146]. Moreover, they would make more frequent use of and rely on health information and services [150], and have the self-efficacy in the ability to understand health information [151] and knowledge [80], which might in turn contribute to the improvement of health literacy [80].

All in all, our current findings acknowledged the importance of social context factors including healthcare practitioners, neighbors, newspapers, television, ethnicity, educational attainment and financial strain that influence health literacy among older adults. Meanwhile, in accordance with previous studies [152,153,154,155,156], our current study suggested the inclusion of social context in health literacy should be highlighted.

## 6. Implications and Limitations

### 6.1. Implications

Due to the influence of health information sources on health literacy, we suggested that healthcare practitioners, neighbors, newspapers and television must be considered as important dissemination patterns and sources of information in health communication. First, the society and company should provide good accessibility and availability for the elderly in interpersonal and mass communication settings in the Chinese context. Especially, the affordability of newspapers and television, and the acceptability barriers of healthcare practitioners and neighbors should be paid more attention. Second, the current findings indicated the influence of enabling factors including educational attainment and financial strain on health literacy. Thus, specific evidence was presented for the reinforcement of health literacy in educational and financial settings. The sustainable and friendly lifelong education and financial support might contribute to facilitating the elderly’s health literacy. Third, focused on the elderly who were ethnic minorities, old-old person, and suffering from chronic diseases, the age-specific, minority-specific and disease-specific measures should be taken to promote health literacy.

### 6.2. Limitations

First, we confessed that the present data had a limitation. Our current data collected in 2017 but submitted for publication in 2022. The last five years has witnessed a great change in society, especially due to the COVID-19 epidemic. There was a lack of consideration to the epidemic effect on health literacy in the present research. Second, the present cross-sectional research could not explore the causal relationship between health information sources and health literacy, and longitudinal research examining health literacy would help to clarify these issues [2]. Third, compared to the number of the usual resident population aged 60 and older in China, the sampling number was relatively small in our research. However, in view of the cost, time, availability and accessibility of collecting the data, the sample size used in our study was 812.

## 7. Conclusions

Health information sources including healthcare practitioners, neighbors, newspapers, and television have a strong influence on health literacy, which implies that the four main types of sources are the important patterns of health information dissemination in the reinforcement of health literacy. In addition, the present findings also indicate age, minority and disease differences in health literacy and confirm the influence of enabling factors including educational attainment and financial strain on health literacy. Based on these findings and their implications, specific evidence is presented for the reinforcement of health literacy in interpersonal and mass communication, and in the educational and financial settings in the Chinese context. The present results also suggest that the age-specific, minority-specific and disease-specific measures should be taken to promote health literacy among older adults.

## Figures and Tables

**Table 1:** Health Literacy Levels by Different Characteristics.

Characteristics		HL Level		
	N (%)	Mean (SD)	F/t	*p*
**Health literacy**	812 (100.0)	56.32 (18.44)	-	-
**Gender**			1.834 ^a^	0.067
Male	379 (46.7)	57.57 (16.87)		
Female	433 (53.3)	55.22 (19.66)		
**Age**			12.467 ^b^	<0.001
60–64	220 (27.2)	61.26 (16.83)		
65–69	187 (23.1)	57.90 (17.93)		
70–74	186 (23.0)	55.56 (18.13)		
75–79	122 (15.1)	53.623 (19.857)		
80+	95 (11.7)	46.46 (17.57)		
**Ethnicity**			7.100 ^a^	<0.001
Han	684 (84.2)	58.59 (16.80)		
Minority	128 (15.8)	44.16 (21.82)		
**Education level**			155.826 ^b^	<0.001
Illiterate	165 (20.3)	36.33 (17.39)		
Primary school	247 (30.4)	52.19 (14.65)		
Junior high school	236 (29.1)	64.85 (12.42)		
Polytechnic school or senior high school	116 (14.3)	69.42 (10.76)		
College and above	48 (5.9)	72.69 (8.68)		
**Occupation**			28.089 ^b^	<0.001
Ordinary staff	56 (6.9)	67.00 (12.13)		
Professionals	154 (19.0)	67.72 (13.18)		
Manager	15 (1.8)	61.47 (14.89)		
Service industry employee	65 (8.0)	58.78 (14.44)		
Production staff	465 (57.3)	51.80 (18.89)		
Others	57 (7.0)	47.74 (17.96)		
**Financial strain**			59.931 ^b^	<0.001
More than enough	48 (5.9)	67.02 (11.62)		
Good enough	287 (35.3)	64.89 (13.60)		
Approximately enough	334 (41.1)	53.68 (17.79)		
Some-what difficult	105 (12.9)	44.75 (18.53)		
Very difficult	38 (4.7)	33.26 (18.12)		
**Self-rated health**			22.074 ^b^	<0.001
Bad	110 (13.5)	48.79 (18.66)		
So-so	280 (34.5)	53.61 (17.54)		
Good	422 (52.0)	60.08 (18.08)		
**Suffering from chronic disease**			0.952 ^a^	0.341
No	130 (16.0)	57.72 (18.30)		
Yes	680 (84.0)	56.04 (18.49)		
**Health information sources**				
Healthcare practitioners			4.694 ^a^	<0.001
No	111 (13.7)	48.78 (17.80)		
Yes	701 (86.3)	57.51 (18.26)		
Family members			3.302 ^a^	0.001
No	368 (45.3)	53.99 (18.84)		
Yes	444 (54.7)	58.25 (17.89)		
Neighbors			6.650 ^a^	<0.001
No	593 (73.0)	54.04 (19.12)		
Yes	219 (27.0)	62.49 (14.79)		
Community workers			1.800 ^a^	0.072
No	670 (82.5)	55.78 (18.46)		
Yes	142 (17.5)	58.85 (18.19)		
Newspapers			13.634 ^a^	<0.001
No	658 (81.0)	53.39 (18.63)		
Yes	154 (19.0)	68.84 (10.80)		
Magazines and books			7.504 ^a^	<0.001
No	748 (92.1)	55.16 (18.27)		
Yes	63 (7.8)	69.98 (14.75)		
Television			10.113 ^a^	<0.001
No	358 (44.1)	49.17 (19.89)		
Yes	454 (55.9)	61.96 (14.98)		
Internet			13.535 ^a^	<0.001
No	733 (90.3)	54.70 (18.47)		
Yes	79 (9.7)	71.30 (9.06)		

Abbreviation: HL represented for health literacy; ^a^ represented for *t* value; ^b^ represented for *F* value.

**Table 2:** Multiple linear regression model I of health information sources factors linked to the elderly’s health literacy.

Predictors	B (95% CI)	β	*p* Value	Tolerance	VIF
Healthcare practitioners	6.180 (2.944, 9.415)	0.115	0.000	0.969	1.032
Family members	3.395 (0.995, 5.795)	0.092	0.006	0.839	1.192
Neighbors	4.564 (1.818, 7.310)	0.110	0.001	0.808	1.237
Community workers	1.998 (−0.933, 4.928)	0.041	0.181	0.966	1.035
Newspapers	10.577 (7.588, 13.567)	0.225	0.000	0.871	1.148
Magazines and books	3.119 (−1.294, 7.532)	0.045	0.166	0.859	1.164
Television	9.510 (7.202, 11.818)	0.256	0.000	0.913	1.095
Internet	9.287 (5.392, 13.182)	0.149	0.000	0.909	1.101
Constant	39.083 (35.571, 42.596)		0.000		
Adjusted R Square	0.259				
F	36.371				
*p*	0.000				

Note: CI, confidence interval; B, non-standardized coefficient; β, standardized coefficient; F represented for *F* value; *p* represented for *p* value. The current multiple linear regression was significant and has strong predictive power, and the multicollinearity was at an acceptable level.

**Table 3:** Multiple linear regression model II of health information sources factors linked to the elderly’s health literacy (controlling demographic variables, socio-economic status and health status).

Predictors	B (95% CI)	β	*p* Value	Tolerance	VIF
**Health information sources**					
Healthcare practitioners	4.577 (2.067, 7.807)	0.085	0.000	0.926	1.080
Family members	1.518 (−0.344, 3.381)	0.041	0.110	0.799	1.252
Neighbors	2.545 (0.434, 4.657)	0.061	0.018	0.782	1.279
Community workers	1.473 (−0.783, 3.729)	0.030	0.200	0.931	1.075
Newspapers	4.280 (1.916, 6.645)	0.091	0.000	0.796	1.256
Magazines and books	1.157 (−2.228, 4.543)	0.017	0.502	0.833	1.201
Television	4.638 (2.811, 6.466)	0.125	0.000	0.834	1.198
Internet	3.008 (−0.157, 6.174)	0.048	0.063	0.794	1.259
**Gender**					
Male (reference)					
Female	−0.129 (−1.878, 1.620)	−0.003	0.885	0.902	1.108
**Age with 5 years increment**	−1.781 (−2.462, −1.101)	−0.129	0.000	0.830	1.205
**Ethnicity**					
Han (reference)					
Minority	−10.005 (−12.362, −7.647)	−0.197	0.000	0.932	1.073
**Education level**					
Illiterate (reference)					
Primary school	11.461 (8.917, 14.005)	0.286	0.000	0.500	2.001
Junior high school	18.016 (15.124, 20.909)	0.443	0.000	0.399	2.507
Polytechnic or senior high school	21.905 (18.410, 25.399)	0.415	0.000	0.460	2.172
College and above	23.433 (18.591, 28.275)	0.297	0.000	0.534	1.872
**Occupation**					
Ordinary staff (reference)					
Professionals	1.372 (−2.482, 5.226)	0.029	0.485	0.303	3.305
Manager	4.873 (−2.252, 11.998)	0.036	0.180	0.742	1.348
Service industry employee	−1.795 (−6.429, 2.839)	−0.026	0.447	0.432	2.315
Production staff	−2.026 (−5.838, 1.787)	−0.054	0.297	0.193	5.175
Others	−2.100 (−7.035, 2.835)	−0.029	0.404	0.437	2.289
**Financial strain**					
More than enough (reference)					
Good enough	0.941 (−3.016, 4.898)	0.024	0.641	0.192	5.215
Approximately enough	−2.971 (−7.074, 1.133)	−0.079	0.156	0.169	5.934
Some-what difficult	−4.168 (−8.870, 0.535)	−0.076	0.082	0.277	3.613
Very difficult	−10.537 (−16.348, −4.726)	−0.121	0.000	0.453	2.206
**Self-rated health**					
Bad (reference)					
So-so	1.117 (−1.644, 3.878)	0.029	0.427	0.400	2.497
Good	2.414 (−0.379, 5.207)	0.065	0.090	0.353	2.833
**Suffering from chronic diseases**	3.430 (0.952, 5.909)	0.068	0.007	0.833	1.201
Constant	34.910 (26.567, 43.253)		0.000		
Adjusted R Square	0.579				
F	42.010				
*p*	0.000				

Note: CI, confidence interval; B, non-standardized coefficient; β, standardized coefficient; F represented for *F* value; *p* represented for *p* value. The current multiple linear regression was significant and has strong predictive power, and the multicollinearity was at an acceptable level.

## Data Availability

The data presented in this study are available on request from the corresponding author. The data are not publicly available due to privacy restrictions.
